# Physiologically Based Biopharmaceutics Model of Apixaban for Biopharmaceutics Risk Assessment

**DOI:** 10.3390/pharmaceutics17030382

**Published:** 2025-03-18

**Authors:** Paulo Paixão, Zvonimir Petric, José A. G. Morais

**Affiliations:** Research Institute for Medicines (iMed.ULisboa), Faculty of Pharmacy, University of Lisbon, 1649-004 Lisboa, Portugal; petric.zvonimir@gmail.com (Z.P.); jagmorais@ff.ulisboa.pt (J.A.G.M.)

**Keywords:** Physiologically Based Biopharmaceutics Modeling, apixaban, model-informed drug development, Critical Biopharmaceutics Attributes

## Abstract

**Background/Objectives:** This study applies a Physiologically Based Biopharmaceutics Modeling (PBBM) framework to predict the bioavailability (BA) and bioequivalence (BE) of apixaban, a borderline BCS Class III/IV drug. It investigates how formulation factors, such as particle size, granulation method, and dissolution conditions, affect apixaban’s in vivo behavior under fasting conditions. **Methods:** A PBBM approach was developed by integrating physicochemical, formulation, and drug-related parameters to simulate dissolution and absorption using a middle-out strategy for combining in silico, in vitro, and in vivo data. The Noyes–Whitney equation was used to predict dissolution influenced by particle size, granulation type, and in vitro dissolution conditions. This information was added to a compartmental absorption model of the gastrointestinal track connected to a classical compartmental model characterizing apixaban’s disposition. **Results:** The study validated the apixaban PBBM predictions by comparing simulated and observed pharmacokinetic profiles across several doses and immediate release formulations (solution and tablets) administered through the oral route. Results demonstrated acceptable prediction accuracy for BA and BE under various conditions. The model’s simulations identified a dissolution safe space, enabling regulatory and development insights into acceptable formulation characteristics. **Conclusions:** These findings highlight the potential of PBBM in streamlining drug development, reducing clinical studies, and supporting regulatory decisions. Specifically, for apixaban, the study demonstrated that particle sizes below 120 µm ensure BE with reference formulations, while formulations with faster dissolution rates, such as smaller particle sizes, align closely with BCS biowaiver criteria. This research emphasizes PBBM as a valuable tool for optimizing drug quality and lifecycle management.

## 1. Introduction

Physiologically Based Biopharmaceutics Modeling (PBBM) has emerged as a powerful tool in pharmaceutical development, offering a mechanistic approach to predicting in vivo drug performance based on in vitro data [1]. By integrating drugs’ physicochemical properties, formulation characteristics, and physiological parameters, PBBM allows for the simulation of absorption and bioavailability (BA) under various conditions. Recent advancements in computational tools and biopharmaceutics modeling highlight the regulatory potential of PBBM in bioequivalence (BE) predictions, biowaivers, and the establishment of dissolution safe spaces, as emphasized in recent EMA and FDA guidance [2,3], along with the ICH M13 series [4].

PBBM’s utility in regulatory contexts is gaining recognition, as these models increasingly inform BE studies and post-approval quality assessments. Regulatory agencies, such as the EMA and the FDA, are adopting model-informed drug development (MIDD) approaches to streamline drug approval processes and reduce the reliance on clinical studies, which has prompted harmonization at the ICH level [5]. PBBM, as part of MIDD, offers a scientific foundation for justifying biowaivers and help in deciding which in vivo studies may need to be performed for the biopharmaceutical characterization of different drug products. In addition, by leveraging PBBM to establish dissolution safe spaces, manufacturers can identify acceptable dissolution profiles that ensure consistent BA despite variability in formulation or production [6]. This aligns with the QbD (Quality by Design) framework, fostering a proactive approach to drug development and lifecycle management.

The implications of PBBM extend beyond initial regulatory applications to enhance the quality and utility of oral drug products throughout their lifecycle. By simulating real-world scenarios, PBBM can assess the impact of changes in production methods, storage conditions, and raw material properties on drug product performance. This capability is invaluable for managing risks associated with post-approval changes and for ensuring the quality of medicines across their lifecycle. For that purpose, PBBM can be employed to evaluate Critical Biopharmaceutics Attributes and inform strategies for maintaining quality and consistency over the drug’s life-use [7].

This capability is particularly significant for drugs like apixaban, a drug with a long-term role in managing thromboembolic disorders, classified as a borderline Biopharmaceutics Classification System (BCS) Class III/IV (high/low solubility and limited permeability) [8]. In fact, apixaban’s borderline pH-independent solubility (with a dose number of 1 at the 10 mg dose) may complicate its BA, emphasizing the need for robust predictive models to assess formulation impacts and ensure consistent in vivo performance.

By being at the limits of the BCS Class III/IV drugs, apixaban biopharmaceutics underscore the importance of understanding critical quality attributes (CQAs) that impact BA and BE. These include the dissolution rate, the API particle size, and the granulation method, which may play pivotal roles in determining the in vivo performance of oral drug products. Apixaban serves as a prime example of the interplay between formulation variables and BA. This study evaluates the impact of dissolution conditions (acidic versus neutral media), production methods (dry versus wet granulation), and particle size variations on apixaban’s performance when administered as immediate release tablets using PBBM. The in silico model integrates these parameters to predict how formulation and manufacturing changes influence BA, providing a mechanistic understanding that can guide development, risk assessment, and regulatory decision making.

This research aligns with ongoing efforts to establish best practices for PBBM implementation in regulatory and industrial settings. Recent workshops and publications by leading scientists and regulatory agencies have emphasized the need for a standardized framework for PBBM applications, including parameterization, validation, and contextualization of model outcomes [1,9,10]. By developing a comprehensive PBBM for apixaban and incorporating dissolution data under varied conditions, production methods, and particle sizes, this study also intends to contribute to the evolving landscape of biopharmaceutics modeling. It underscores the importance of these models in predicting critical BA attributes, supporting regulatory decisions, and fostering innovation in drug development.

## 2. Materials and Methods

### 2.1. Available Literature Data on Apixaban

Apixaban is a non-ionizable molecule (MW of 495.5 g/mol) with a water solubility of 40 µg/mL and a Caco-2 permeability of 0.9 × 10^−6^ cm/s [11]. Its highest single recommended single dose is 10 mg, being thus considered a borderline BCS Class III drug (dose number of 1) [8]. The data available in the literature [12] include the dissolution profiles (Type I apparatus, 75 rpm, 900 mL, in pH 6.8 with 0.05% SDS or 0.1 N HCl media) of several oral tablet formulations differing in their production process (dry vs. wet granulation), particle size (D90 from 10 µm to 120 µm), and dosage strength (2.5 mg to 20 mg), with the drug formulated in the crystalline form. It was also concluded that the formulations used in the general clinical studies were made through dry granulation, with an apixaban crystal particle size of 83 µm. In vivo data were available after the single dose intravenous administration of apixaban 5 mg to 20 healthy subjects [13]. In vivo data were also available after the oral administration of apixaban’s doses of 0.5 mg, 1.0 mg, and 2.5 mg as solution to groups of 6 healthy subjects and apixaban’s doses of 2.5 mg, 5 mg, 10 mg, 25 mg, and 50 mg as immediate release tablets for 6 to 12 healthy subjects after fasting using single dose administration [14,15]. BE study results from two non-bioequivalent formulations are also available [12]. Data were gathered from existing tables or extracted from available plots using PlotDigitizer (version 2.6.9).

### 2.2. Dissolution Model of Apixaban

For the dissolution characterization of the different formulations included in [12], a population modeling approach was developed using the parametric maximum likelihood solution via the expectation–maximization algorithm (MLEM) in ADAPT 5 [16] to fit the Noyes–Whitney semi-mechanistic equation to the dissolution data as the base model and the dose (5 mg vs. 20 mg), the production process (wet granulation vs. dry granulation), the dissolution media (pH 6.8 with 0.05% SDS vs. 0.1 N HCl), and the particle size (10 µm to 120 µm) as covariates according to the following equations:

(1) $$\left\{\begin{array}{c} \frac{dSolid}{dt}=-Z\frac{3\times CD}{ro\times h\times Size}\times Solid\times \left(Cs-\frac{Soluble}{V}\right)\\ \frac{dSoluble}{dt}=+Z\frac{3\times CD}{ro\times h\times Size}\times Solid\times \left(Cs-\frac{Soluble}{V}\right) \end{array}\right.$$

where Z = Zwet + A × Zdry + B × Z_HCl_ with Zwet is the Zvalue for the wet granulation formulations, Zdry is the added Z value for the dry granulation process, and Z_HCl_ is the added Zvalue for the HCL dissolution media. A and B are the activators (0 vs. 1) of dry granulation and the 0.1 N HCl dissolution media, CD is the diffusion coefficient with a value of 0.018 cm^2^/h, ro is the density of the drug with a value of 1200 mg/cm^3^, h is the diffusion layer thickness with a value of 0.003 cm, and Size is the variable particle size (cm) of the drug crystals [17,18]. Solid and soluble are the amounts of apixaban in each of the respective physical forms.

A total of 16 average dissolution profiles were available, including full dissolution profiles for 20 mg wet and dry granulation tablets (particle size: 83.9 µm) in either pH 6.8 with 0.05% SDS or 0.1 N HCl dissolution media. Additionally, full dissolution profiles were available for 5 mg wet granulation tablets with particle sizes of 50 µm or 89 µm, also for pH 6.8 with 0.05% SDS or 0.1 N HCl dissolution media. Furthermore, eight single dissolution time points (30 min) were recorded for 5 mg dry granulation tablets with particle sizes ranging from 11 µm to 129 µm in pH 6.8 with 0.05% SDS, resulting in a total of 58 individual data points.

### 2.3. Disposition Model of Apixaban

The in vivo disposition was modeled by fitting a compartmental model to the mean IV profiles obtained after the administration of 5 mg of apixaban to healthy subjects, available in [13], using the maximum likelihood individual estimation module in ADAPT 5 [16]. Two mamillary models, including either 2 or 3 compartments, were tested, and selection was made based on the Aikaike selection criteria.

### 2.4. Optimization of the Physiologically Based Biopharmaceutics Model for Oral Formulations of Apixaban

**Figure 1:**
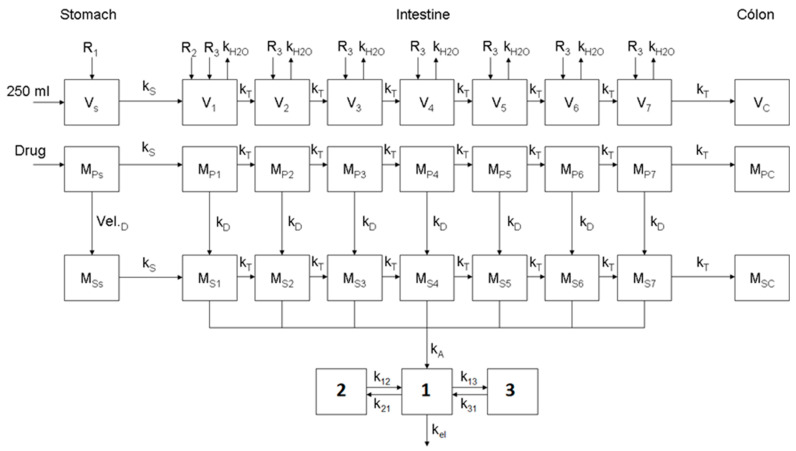
Representation of the oral PBPK model of apixaban.

The oral apixaban model, developed using the PBBM framework, based on previous works [18,19], was implemented using the Berkeley–Madonna software package (version 8.3.18) and is depicted in Figure 1. Namely, it consists of a physiological representation of the water contents in the gastrointestinal tract (GIT) and the movement of the drug in both particles and soluble forms. Specifically, the GIT was modeled as three segments, each comprising a series of multiple compartments connected by linear transfer kinetics. The first segment represents the stomach, a single compartment with negligible absorption, connected to the second segment, which represents the small intestine and is divided into a sequence of seven compartments. The residence time in each compartment was calculated based on the physiological length of the respective GIT segment, assuming a constant transit across the intestine. Furthermore, a linear decrease in the intestine radius from 1.75 cm at the proximal end to 1 cm at the distal end was also assumed. Finally, the last segment, represented as a single compartment, corresponds to the colon, where absorption is considered negligible.

The drug (apixaban), administered as a solution or an immediate release dosage form (tablet), was introduced directly into the stomach with 250 mL of water. Upon disintegration (assumed to occur instantaneously), the solid (M_p_) and soluble mass of the drug (M_s_), moved out of the stomach according to the gastric emptying rate (k_s_), reaching the subsequent compartment and migrating through the different intestinal compartments according to their respective transit rates (k_T_). All transits processes were modeled as first order kinetics (assumption). To determine the concentration of the dissolved drug, the water content (V_i_) in the GIT was modeled using a parallel physiological model based on the previously described segmented series. Water volume was assumed to depend on salivary and gastric (R_1_), duodenal (R_2_), and intestinal mucous (R_3_) secretion rates, as well as the intestinal water reabsorption process (k_H2O_). The drug dissolution rate (k_D_) of each compartment was defined by the Noyes−Whitney equation, without assuming “sink conditions”. Apixaban absorption was characterized as a passive diffusion mechanism, based on its Caco-2 permeability and extrapolation to an effective permeability (Peff) according to the equation log(Peff)(cm/h) = 0.932 + 0.763 × log(Papp)(cm/h) + 0.0324 × RBN, where RBN represents the number of rotatable bonds in the molecule [18]. Consequently, apixaban absorption was modeled as a first order process, with the absorption rate constant (ka) calculated as ka = (2Peff)/radius compartment.

By quantifying the mass of solid drug reaching the colon or the mass of drug being absorbed, it is possible to determine the BA limited by dissolution (F_d_) and absorption (F_abs_) and as absolute BA (F_oral_). After absorption, the disposition was modeled using a standard multi-compartmental method, parametrized on the intravenous data by fitting. Also, according to available PK data, apixaban’s total and renal clearances are 3.3 L/h and 0.9 L/h, respectively [8]. This corresponds to a (very) low hepatic extraction ratio of 0.039, and as a result, the first-pass effect was excluded from the final model.

A “middle-out” approach was adopted for the characterization of the apixaban’s parameters in the model. In general, disposition parameters (see Table 1) were fitted to the IV plasma profile obtained after the single dose of intravenous administration of apixaban 5 mg and used directly in the disposition part of the model in Figure 1. Intestinal permeability, and apixaban’s solubility were determined from independent data collected in vitro, as previously described. For all solid formulations, the dissolution profiles considered biorelevant were those simulated in 0.1 N HCl, as in this medium no surfactant was used and apixaban exhibits no pH-dependent solubility. Consequently, and based on the fixed parameters obtained from the PopPK model describing the in vitro dissolution of apixaban, a Z value of 31 was considered in the simulations for dry granulation formulations used in studies from [14,15], while a Z value of 22.3 was applied for wet granulation formulations described in the BE study [12]. Particle size for the tablets for the data from [14,15] was considered 83 µm, and it was either 50 µm or 89 µm in the BE study [12]. The drug parameters introduced in the model, as well as their origins, are presented in Table 1. No further parameter optimization was made in order to better fit simulations to the in vivo data. In fact, these final parameters were validated by simulating the PK profiles after the administration of apixaban as a solution in 3 different doses (0.5 mg, 1 mg, and 2.5 mg), as tablets under 5 different doses (2.5 mg, 5 mg, 10 mg, 25 mg, and 50 mg), and in a BE study with two formulations that do not meet the BE criteria in Cmax (T/R of 86.3% and an 90% CI of 78.8–93.9%), all to healthy subjects under fasting conditions. Final agreement between the observed and simulated results was made by calculating the prediction error (PE), defined as %PE = [(observed value − predicted value)/observed value] × 100, for each of the main PK exposure metrics, Cmax, and AUC_0-t_, as well as for Tmax.

### 2.5. Simulations Based on the Physiologically Based Biopharmaceutics Model of Apixaban

The optimized final PBBM was used for simulating different formulations in order to define a safe space of dissolution as well as to define the risks of the current biowaiver approaches accepted for BCS Class III drugs. To this end, simulations were made for the administration of 5 mg of apixaban as a solution and as tablets formulated through dry granulation process with particle sizes from 10 µm up to 210 µm. BE for the mean profile was determined by taking both the solution and the 83 µm formulation as comparators.

Simulations of the dissolution profiles, obtained using apparatus II at 75 rpm in 0.1 N HCl, conditions closely aligning with regulatory biowaiver dissolution requirements, were also conducted for the same tablets (i.e., produced via dry granulation), with particle sizes ranging from 10 µm to 210 µm.

## 3. Results

### 3.1. Dissolution Model of Apixaban

**Figure 2:**
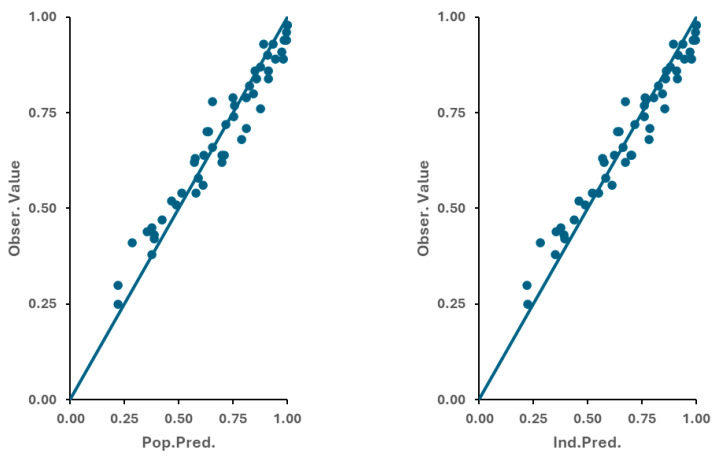
Goodness-of-fit (GOF) plots of the dissolution model.

The data used in the dissolution model included both multiple time points’ and single time points’ dissolution profiles. For each situation, information was available on the percentage dissolved at a particular time point (10 min to 60 min), the dissolution media (0.05% SLS in 50 mM phosphate pH 6.8 buffer vs. 0.1 N HCl), the type of granulation process (wet vs. dry), as well as the API particle size D90 (from 10 µm to 120 µm). The role of the SDS in the dissolution media was described as a wetting aid rather than to increase the solubility of apixaban, and thus the solubility value considered in the model was kept constant, with a value of 40 µg/mL. As such, only the Z-factor was estimated in the model while considering the type of granulation and the dissolution media as covariates explaining its variability. The residual error was assumed to be additive. Final results (−2logLikelihood = −150.925) showed that the Z parameter under the dissolution media pH 6.8 with SDS had a value of 44.8 if a wet granulation process was used or 53.4 if a dry granulation process was used. These values are reduced by −22.5 if the dissolution medium 0.1 N HCl is considered. Goodness-of-fit (GOF) plots of the results over the final dissolution model are presented in Figure 2. As it can be observed, some bias is present, indicating an oversimplification of the model. However, as there is still good agreement between the observed and predicted values, for the sake of simplicity and mechanistic interpretation, the obtained Z parameter was further used.

### 3.2. Disposition Model of Apixaban

**Figure 3:**
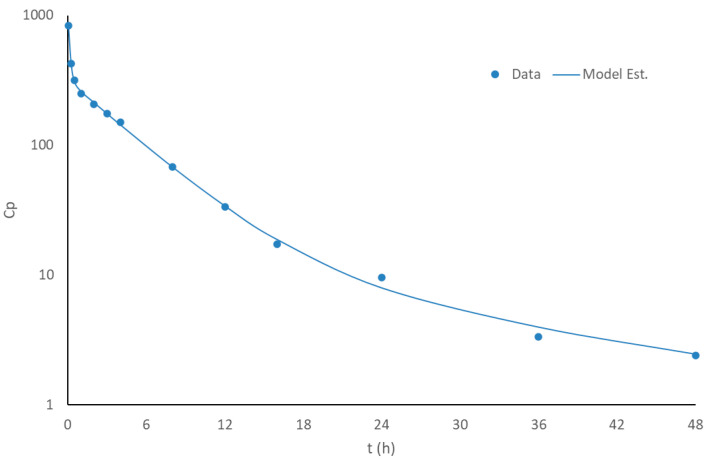
Fitting of a 3-compartment model to the plasma data following intravenous administration of 5 mg of apixaban in healthy volunteers.

Regarding the fitting of a compartmental model to the published mean profile observed after the intravenous administration of 5 mg of apixaban to 20 healthy subjects [13], a three-compartmental model (r^2^ = 0.999) showed a better description than a two-compartment one (r^2^ = 0.998), with a reduced AIC criterium (82.54 vs. 84.17). The results of the fitting of the three-compartment disposition model, as well as the fitted parameters, can be seen in Figure 3 and Table 1. There is good agreement between the observed and predicted values, and thus the model parameters were then used for characterizing the disposition part of the PBBM model.

### 3.3. Optimization of the Physiologically Based Biopharmaceutics Model of Apixaban

Simulations were performed using the drug parameters described in Table 1. The simulations for the solutions were made in order to validate the overall drug absorption part of the PBBM and to validate the disposition PK part.

**Figure 4:**
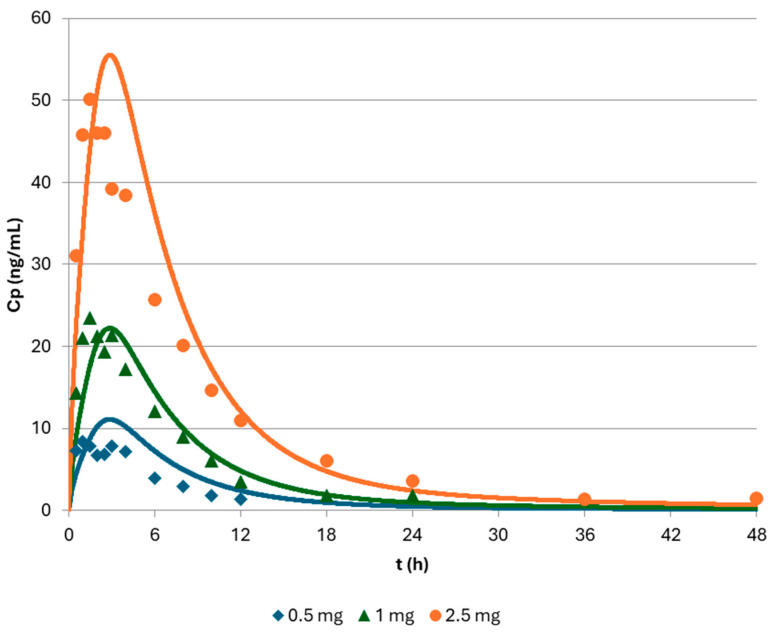
Predicted plasma profiles of various oral solution doses of apixaban (0.5 mg, 1 mg, 2.5 mg), as simulated by the physiologically based biopharmaceutics model of apixaban.

Simulation results of different doses of drug administered orally in solution (0.5 mg, 1 mg, and 2.5 mg) to groups of six healthy subjects are depicted in Figure 4, and the agreement between the observed and simulated PK results are in Table 2. Good agreement is seen in the overall profiles, with similar Cmax and AUC but slightly delayed predicted Tmax. This may be because PBBM gastric emptying was optimized for particles in solution and not for solutions themselves. The disposition part seems to be well-captured by the PBBM, as the latter simulated concentrations are similar to the ones observed in vivo. Based on the model predicting the absorbed mass after the administration of 2.5 mg through oral solution, the oral BA of the solution was 52%, similar to what is described for apixaban [8]. The prediction errors are less than 15% for the 1 mg and 2.5 mg doses for Cmax and AUC. Slightly higher values were observed for the 0.5 mg formulation. The low number of subjects included in this study should be taken into consideration (n = 6 in each group), and also the fact that most of the 0.5 mg dose concentrations were close to the lower limit of quantification of the used analytical method (1 ng/mL) [14].

**Figure 5:**
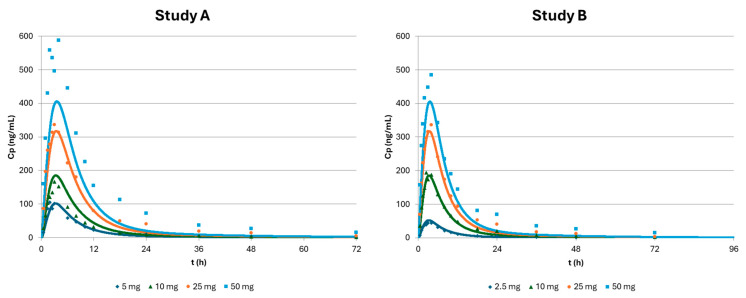
Predicted plasma profiles of various apixaban tablets across different doses (2.5 mg, 5 mg, 10 mg, 25 mg, and 50 mg) as simulated by the physiologically based biopharmaceutics model of apixaban compared with in vivo independent studies A and B.

The simulation results for apixaban tablets administered orally (in various doses) are presented in Figure 5, and the agreement between the observed and simulated PK exposure metrics is shown in Table 3. Two independent studies were available with similar protocols and analytical methods but in different groups of healthy subjects. In the first study, A [14], each studied group included 6–7 subjects, whereas in the second study, B [15], each group included 12 Caucasian subjects. Overall, good agreement between the model’s prediction and the observed profiles is seen, with similar Cmax and Tmax values. Again, the disposition part seems to be well-captured by the PBBM for the lower doses (<10 mg), as the latter simulated concentrations are similar to the ones observed in vivo. However, that is not the case for doses higher than 10 mg, where the concentrations after 24 h all seem to be underpredicted. This may explain some underpredictions observed in the AUC for these higher doses. Based on the model of the predicted dissolved drug and the absorbed mass after the administration of 2.5 mg as a tablet, 99% of the drug is dissolved when in the intestine, and the oral BA was 48%, which is, again, close to what is described for apixaban [8]. For higher doses, e.g., 25 mg, only 63% of the dose is able to be dissolved in the intestine, and the oral BA is reduced to 31%, in line with a mass balance study with a dose of 20 mg [20] where the PBBM predicts an absorption of 35% and a mass balance study of around 43%. The prediction errors are generally less than 15% for all but the 50 mg dose in all PK metrics, showing an overall good prediction ability. For the 50 mg dose, where PE% as high as 42% was obtained, this may be due to the disposition part not being fully in line with the observed latter concentrations.

A final validation was made by simulating the in vivo profiles of two tablets produced through a wet granulation process with different in vitro dissolution profiles due to different particle sizes of 50 µm and 89 µm, as provided in [12]. Although the in vitro data were clearly described, regarding the in vivo BE study, only the Cmax and AUC values were made available, as well as the 90% CI of the T/R comparison for Cmax. No sample size was provided. Results of the comparisons between the simulated profiles and the observed PK metrics are visible in Table 4. As it can be seen, the %PE is lower than 10% for Cmax and lower than 17% for AUC. The conclusions of BE are similar for the in vivo and the simulated data.

**Figure 6:**
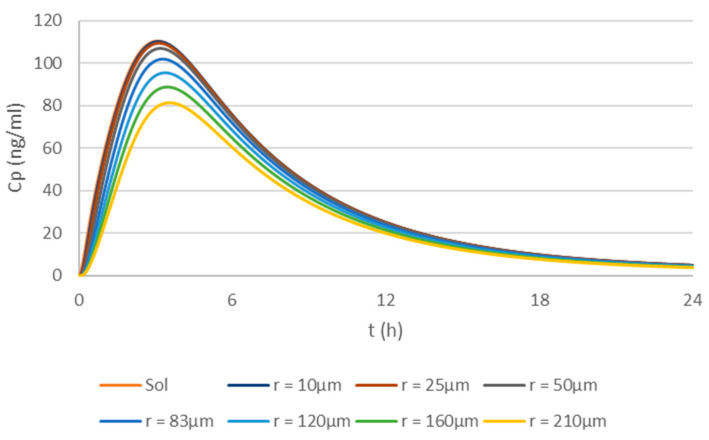
Simulations of the plasma profiles for the 5 mg apixaban solution and dry granulation tablets with particle sizes increasing from 10 µm to 210 µm based on the dissolution simulated profiles shown in Figure 7.

**Figure 7:**
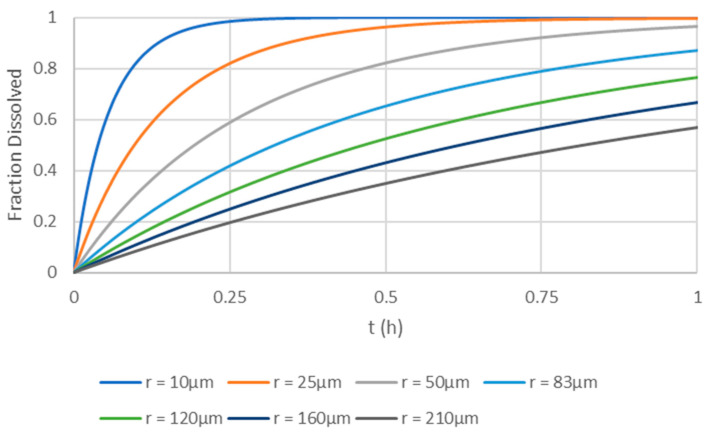
Simulations of the dissolution profiles (apparatus II, 75 rpm, 0.1 N HCl) for the 5 mg dry granulation tablets with particle sizes increasing from 10 µm to 210 µm used for the plasma PK profile simulations presented in Figure 6.

For the simulations based on the physiologically based biopharmaceutics model of apixaban, the simulations of the plasma profiles for the 5 mg apixaban solution and the dry granulation tablets with particle sizes increasing from 10 µm to 210 µm are presented in Figure 6. The ratio of the different formulations to the solution (Reference 1) or to the dry granulation 83 µm formulation (Reference 2) can be seen in Table 5. The dissolution profiles in apparatus II, 75 rpm, 0.1 N HCl, simulated according to the Noyes–Whitney mechanistic equation, can be seen in Figure 7.

The simulations showed that the 83 µm formulation and the solution are bioequivalent. BE in the solution, defined as the T/R ratio being inside of the 80–125% interval, can be observed up to particle sizes of 160 µm. Regarding the reference tablet of 83 µm, BE can be shown by formulations with particle sizes up to 210 µm. Regarding dissolution, a formulation with particle sizes up to 20–25 µm would result in very fast dissolutions, with more than 85% dissolved at 15 min. The 160 µm formulation, with only 67% dissolved at 60 min, would not even been considered a fast dissolving one. In these conditions, the 210 µm would only result in 57% dissolved apixaban at 60 min.

## 4. Discussion

In this paper, we describe the development and application of a Physiologically Based Biopharmaceutical Model for apixaban. Apixaban is an oral direct factor Xa inhibitor that inhibits both free and clot-bound factor Xa, and it has been approved for clinical use in several thromboembolic disorders [8]. Despite being a BCS Class III drug and, in principle, an easy drug to formulate, it is only a borderline one, and, according to its development process, its dissolution is not very fast [12]. Thus, when establishing BE, it is not subject to a BCS biowaiver approach, being a good example for testing the BCS biowaiver concept itself based on this reason alone. Also, due to the vast in vitro dissolution data associated with in vivo clinical pharmacokinetic data, it is also a good example for examining the use of PBBM for understanding critical quality attributes.

In the modeling exercise based on the PBBM framework, dissolutions of several formulations with different quality characteristics and different dissolution conditions that were available in the literature were modeled together through a population modeling approach using the Noyes–Whitney mechanistic equation. The model included the particle size, the wet vs. dry granulation process, and the dissolution conditions as co-variables explaining the overall variability of the data. There are several established methods, such as direct dissolution input, the Johnson model, the Z-factor, the P-PSD model, and the diffusion layer model, to name a few, that can be utilized for integrating dissolution data in PBBM [10]. In our model, we used a version of the Z-factor approach that has its strengths based on the fact that, although simplified, it is based on a mechanistic framework. However, several aspects that can confound particle dissolution, such as disintegration and coning, are not taken into consideration in this dissolution model and are seen as one of its limitations [21]. Those factors could explain some of the bias present in the model’s fitting to the dissolution data, which could eventually be improved by including an additional disintegration step in the model. However, this kinetic moment would only be physiologically relevant in the gastric compartment and, because disintegration of IR products is usually very fast, would probably have limited relevance in the remaining areas of the gastrointestinal tract. Thus, it was not further explored.

PBBM optimization was performed through a middle–out approach by combining bottom–up in vitro predictions with limited top–down fitting of key model parameters for clinical data, as performed by others with success in developing reliable PBPK models [22]. In this case, the only top–down fitting to clinical data was performed on the disposition part of the model and based on plasma profiles after IV administration. As such, all of the remaining absorption parameters of the model were obtained through bottom–up in vitro or in silico prediction. This type of approach has been highlighted as the preferred approach for model development by regulatory authorities [9].

Regarding validation, this was performed following the typical regulatory requirements by assessing the ability of the model to predict the range of the observed outcome of representative in vivo pharmacokinetic studies, e.g., different dose levels of different types of formulations under single dose administrations, as well as in a BE study with two formulations not able to show BE [2]. This latter assessment is considered a major one in increasing confidence in the model [3]. The ability of the model to predict plasma profiles was assessed through determination of the Cmax, Tmax, and AUC and their corresponding prediction errors. In this regard, acceptance values are typically dependent on the regulatory risk of the modeling exercise. If, for high-risk situations where BE studies are to be waived or to evaluate whether a dissolution method is bio predictive, usually, the predicted systemic exposure should be comparable (<±10–15%) to the observed in vivo PK data [3,23]. In our validation, and regarding the prediction of the apixaban profiles after administration as a solution, we observed that these requirements could be obtained for the 1 mg and 2.5 mg doses, but not the 0.5 mg dose. This may be due to the low concentrations observed for the 0.5 mg dose and some limitations in their determination, as the lower limit of the analytical method (1 ng/mL) was less than 10% of the observed average Cmax for this dose. This, together with the low number of subjects studied (n = 6), can justify the observed %PE in the order of 30–40%. Regarding the apixaban’s tablet formulations, prediction errors were acceptable but varied between studies. For example, for the 10 mg formulation in study A [14], prediction errors were −10% and −24% for Cmax and AUC, respectively, not complying with the stricter regulatory demands. However, for study B [15], the same were 5% and 13%, respectively, all below the ±15% acceptance error. This discrepancy highlights the challenges of validation if the focus is on a single study rather than evaluating the model’s performance across multiple datasets. It should be mentioned that both studies used the same analytical method, the same type of population, and similar clinical protocols but different clinical facilities.

Another interesting finding is that although Cmax was relatively well-predicted in all doses, AUC showed increasingly worse predictions for the 25 mg and 50 mg higher doses. Apixaban exposure is known to increase proportionally with doses up to 10 mg, but at doses ≥ 25 mg, less-than-proportional increases are observed [8]. In our model, we have assumed that the drug’s permeability was based on a first order rate process. In fact, apixaban is described as a substrate of P-GP, and relevant interactions are observed when administered with either ketoconazole (CYP3A4/P-GP strong inhibitor) or Rifampin (CYP3A4/P-GP strong inducer), and their co-administration is not recommended. However, no dose adjustment of apixaban is required when administered with agents that are not considered strong inhibitors of both CYP3A4 and P-GP. Also, when observing the non-linearity for higher doses, we see a reduction in the BA. If the problem was related to P-GP saturation, then an increase in BA would be expected. Taking all into consideration, the assumption of a passive first order process in the absorption of apixaban is still acceptable in the present biopharmaceutical context. In another line of reasoning, the elimination half-life of apixaban was increased for higher doses in several studies [13,14,15]. This may be due to saturation on one of the multiple metabolic pathways or an increase in bile excretion with possible enterohepatic recirculation, which, although usually insignificant, can increase for concentrations above 200 ng/mL [20]. Both of these processes are probably not relevant in the IV data that were obtained with a dose of 5 mg. In fact, by considering the disposition of apixaban as the one observed for the 5 mg IV dose, all of the possible non-linear processes were not considered in the PBBM. Although this may indicate a simplification of the final model, the lack of IV data at higher doses did not allow us to define a disposition model accounting for these non-linear effects. However, because this is only seen for higher, not clinically relevant doses and is not related to the absorption process itself, it does not invalidate the use of the model for biopharmaceutical purposes using clinically relevant (lower) doses. This is further supported by the similar conclusion observed between the in vivo BE study and the PBBM BE simulations obtained with a dose of 5 mg.

In this study, (virtual) BE assessment was performed only based on the mean plasma profile simulated without considering variability in either the physiological or drug-related parameters. As such, no questions regarding the number of subjects included in the virtual simulations or the need to compare 90% confidence intervals were considered. In fact, the inclusion of both between-subject and within-subject variability is still a matter of debate due to lack of sufficient information [9]. However, by simulating a single plasma profile, it is worth questioning whether a 20% difference threshold for BE remains appropriate. In fact, if point estimates are considered, FDA data on BE studies from a 12-year period revealed that in nearly 98% of studies, the AUC of a generic product differed from that of the innovator product by less than 10% [24]. Thus, considering a 10% difference in AUC or Cmax between two different drug product simulations as a cut-off point for BE may be a more suitable simulation-based approach. Taking this consideration for the proposed simulations, it could be seen that if the BCS concept was considered, formulations with particle sizes of up to 25 µm resulted in very fast in vitro dissolutions, with more than 85% of the drug dissolved by 15 min (Figure 7) and a plasma profile basically equal to the administration of the drug as a solution (Figure 6 and Table 5). If the particle size is increased to 50 µm, the in vitro dissolution would still be considered fast, with more than 85% of the drug dissolved by 30 min, and the product would still be considered BE with the oral solution. In fact, the 83 µm particle size formulation is still BE to the oral solution despite the dissolution only being complete (more than 85% of the drug dissolved) very close to the 1 h dissolution time. This result is in line with previous simulations using a commercial PBBM software [25], and it supports the current regulatory framework for BCS biowaivers [26] by providing a conservative in vitro based dissolution waiver. However, it also opens the door for the possibility of accepting the same dissolution conditions for BCS Class III drugs as requested for BCS Class I drugs in the future.

Regarding the 83 µm particle size formulation, all formulations up to a size of around 120 µm are expected to be BE with it (Table 5). This means that regarding the dissolution space, all formulations showing more than 65% at 1 h dissolution time in apparatus II, 75 rpm, and 0.1 N HCl should be BE. If considering the QC method performed in apparatus II, 75 rpm, with 0.05% SLS in 50 mM phosphate pH 6.8 buffer as dissolution media, then a dissolution of more than 70% at 30 min or more than 85% at 45 min should also ensure that the products are BE. Under a drug development context, this information could be used either for setting the dissolution conditions for batch release, if the drug product is already approved, or to help develop a generic formulation with increased confidence that will result in a BE for the required in vivo studies.

## 5. Conclusions

The presented apixaban model based on the PBBM framework was based only on publicly available data that allowed for the development of an oral absorption PBPK model, which was optimized through a “middle-out” approach. In vitro data (Caco-2 permeability, in vitro dissolution, water solubility) were used to describe the drug’s in vivo dissolution and absorption process. In vivo IV PK data were used to describe the in vivo disposition process. The final model showed acceptable in vivo predictability for both oral solutions and tablets at different doses. This model may be able to help the formulation development process by identifying and in silico testing the main constraints in drug absorption.

## Figures and Tables

**Table 1:** Drug-specific parameters incorporated into the physiologically based biopharmaceutics model of apixaban along with their original source values.

Parameter	Simulations			
	Solution [14,15]	Tablets [14,15]	BE Study [12]	Reference
Dose (mg)	0.5, 1, 2.5	2.5, 5, 10, 25, 50	5	-
Absorption				
Peff (cm/h)		1.57 × 10^−1^		in vitro [11,18]
Solubility (µg/mL)		40		in vitro [11]
Dissolution				
Z	-	31	22.3	in vitro fitting [12]
Particle size (µm)	-	83	50/89	in vitro [12]
Disposition				
K_10_ (h^−1^)		0.5409		in vivo fitting [13]
k_12_ (h^−1^)		0.086		in vivo fitting [13]
k_21_ (h^−1^)		0.0441		in vivo fitting [13]
k_13_ (h^−1^)		4.319		in vivo fitting [13]
k_31_ (h^−1^)		2.24		in vivo fitting [13]
V_1_ (L)		4.756		in vivo fitting [13]

**Table 2:** Comparison of in vivo exposure metrics (Cmax and AUC) and Tmax with simulated values derived from the physiologically based biopharmaceutics model of apixaban for the oral solution doses (0.5 mg, 1 mg, 2.5 mg).

	0.5 mg			1 mg			2.5 mg		
	Obs	Pred	%PE	Obs	Pred	%PE	Obs	Pred	%PE
C_max_ (ng/mL)	8.42	11.09	−32	23.46	22.19	5	50.15	55.46	−11
T_max_ (h)	1.00	3.00	−200	1.50	3.00	−100	1.50	3.00	−100
AUC (ng·h/mL)	54.10	78.06	−44	176.32	197.88	−12	454.96	494.69	−9

**Table 3:** Comparison of in vivo exposure metrics (Cmax and AUC) and Tmax with simulated values derived from the physiologically based biopharmaceutics model of apixaban tablets in study A and study B.

**Study A**	**5 mg**			**10 mg**			**25 mg**			**50 mg**		
	**Obs**	**Pred**	**%PE**	**Obs**	**Pred**	**%PE**	**Obs**	**Pred**	**%PE**	**Obs**	**Pred**	**%PE**
C_max_ (ng/mL)	105.68	101.20	4	166.54	183.75	−10	336.84	312.63	7	587.95	401.38	32
T_max_ (h)	2.00	3.00	−50	3.00	3.00	0	3.00	3.00	0	4.00	4.00	0
AUC (ng·h/mL)	982.48	906.13	8	1347.37	1667.98	−24	3658.83	2961.29	19	6759.43	3906.82	42
**Study B**	**2.5 mg**			**10 mg**			**25 mg**			**50 mg**		
	**Obs**	**Pred**	**%PE**	**Obs**	**Pred**	**%PE**	**Obs**	**Pred**	**%PE**	**Obs**	**Pred**	**%PE**
C_max_ (ng/mL)	44.21	51.74	−17	194.09	183.75	5	335.98	312.63	7	484.65	401.38	17
T_max_ (h)	4.00	3.00	25	2.50	3.00	−20	4.00	3.00	25	4.00	4.00	0
AUC (ng·h/mL)	451.03	461.70	−2	1917.80	1667.98	13	3653.04	2961.29	19	5681.64	3906.82	31

**Table 4:** Comparison of the observed (in vivo) exposure metrics (Cmax and AUC) from the BE study with predicted exposure metrics simulated by the physiologically based biopharmaceutics model of apixaban for two different oral tablets.

	5 mg/50 µm			5 mg/89 µm			BE		
	Obs	Pred	%PE	Obs	Pred	%PE	Obs	Pred	%PE
Cmax (ng/mL)	101.80	104.23	−2	87.80	94.93	−8	0.86	0.91	−6
AUC (ng∙h/mL)	1088.00	938.55	14	1030.00	856.75	17	0.95	0.91	4

**Table 5:** The T/R ratio of the different formulations (T) to the solution (R1) and to the dry granulation 83 µm formulation (R2).

**T/R_1_ Ratio**	**Solution**	**10 µm**	**25 µm**	**50 µm**	**83 µm**	**120 µm**	**160 µm**	**210 µm**
C_max_	1.00	1.00	0.99	0.97	0.92	0.86	0.80	0.74
T_max_	1.00	1.01	1.02	1.04	1.06	1.10	1.12	1.15
AUC	1.00	1.00	0.99	0.96	0.91	0.86	0.80	0.74
**T/R_2_ Ratio**	**Solution**	**10 µm**	**25 µm**	**50 µm**	**83 µm**	**120 µm**	**160 µm**	**210 µm**
C_max_	1.08	1.08	1.07	1.05	1.00	0.94	0.87	0.80
T_max_	0.94	0.95	0.95	0.97	1.00	1.03	1.05	1.08
AUC	1.09	1.09	1.08	1.05	1.00	0.94	0.88	0.81

## Data Availability

The original contributions presented in this study are included in the article. Further inquiries can be directed to the corresponding author.

## Abbreviations

The following abbreviations are used in this manuscript:

AIC	Akaike Information Criterion
API	Active Pharmaceutical Ingredient
AUC	Area Under the Curve
BA	Bioavailability
BCS	Biopharmaceutics Classification System
BE	Bioequivalence
CI	Confidence interval
Cmax	Maximum Plasma Concentration
CQAs	Critical quality attributes
EMA	European Medicines Agency
FDA	U.S. Food and Drug Administration
GIT	Gastrointestinal tract
GOF	Goodness-of-fit
ICH	International Council for Harmonisation of Technical Requirements for Pharmaceuticals for Human Use
IV	Intravenous
MIDD	Model-informed drug development
Obs	Observed
PBBM	Physiologically Based Biopharmaceutics Modeling
PBPK	Physiologically Based Pharmacokinetic
%PE	Prediction error
Peff	Effective permeability
Pred	Predicted
PK	Pharmacokinetics
QbD	Quality by Design
Tmax	Time to Reach Maximum Plasma Concentration
